# Pre-treatment with P2Y12 inhibitors in acute myocardial infarction with cardiogenic shock

**DOI:** 10.1093/ehjcvp/pvaf019

**Published:** 2025-04-01

**Authors:** Anthony Elhadad, David Sulman, Niki Procopi, Perrine Devos, Frederic Beaupré, Nassim Braik, Louis Giovachini, Pierre Charleux, Alain Combes, Juliette Chommeloux, Patrick Ecollan, Stéphanie Rouanet, Mathieu Kerneis, Johanne Silvain, Gilles Montalescot, Michel Zeitouni

**Affiliations:** Sorbonne Université, ACTION Study Group, INSERM UMRS 1166, Institut de Cardiologie, Hôpital Pitié-Salpêtrière (AP-HP), Paris 75013, France; Sorbonne Université, ACTION Study Group, INSERM UMRS 1166, Institut de Cardiologie, Hôpital Pitié-Salpêtrière (AP-HP), Paris 75013, France; Sorbonne Université, ACTION Study Group, INSERM UMRS 1166, Institut de Cardiologie, Hôpital Pitié-Salpêtrière (AP-HP), Paris 75013, France; Sorbonne Université, ACTION Study Group, INSERM UMRS 1166, Institut de Cardiologie, Hôpital Pitié-Salpêtrière (AP-HP), Paris 75013, France; Sorbonne Université, ACTION Study Group, INSERM UMRS 1166, Institut de Cardiologie, Hôpital Pitié-Salpêtrière (AP-HP), Paris 75013, France; Sorbonne Université, ACTION Study Group, INSERM UMRS 1166, Institut de Cardiologie, Hôpital Pitié-Salpêtrière (AP-HP), Paris 75013, France; Sorbonne Université, ACTION Study Group, INSERM UMRS 1166, Institut de Cardiologie, Hôpital Pitié-Salpêtrière (AP-HP), Paris 75013, France; Sorbonne Université, ACTION Study Group, INSERM UMRS 1166, Institut de Cardiologie, Hôpital Pitié-Salpêtrière (AP-HP), Paris 75013, France; Sorbonne Université, Institut de Cardiologie, Service de Médecine Intensive-Réanimation Hôpital Pitié-Salpêtrière (AP-HP), Paris 75013, France; Sorbonne Université, Institut de Cardiologie, Service de Médecine Intensive-Réanimation Hôpital Pitié-Salpêtrière (AP-HP), Paris 75013, France; Sorbonne Université, Institut de Cardiologie, service d'accueil des urgences, SMUR, Hopital Pitié-Salpêtrière (APHP), Paris 75013, France; Statistician Unit, StatEthic, ACTION Study Group, Levallois-Perret, France; Sorbonne Université, ACTION Study Group, INSERM UMRS 1166, Institut de Cardiologie, Hôpital Pitié-Salpêtrière (AP-HP), Paris 75013, France; Sorbonne Université, ACTION Study Group, INSERM UMRS 1166, Institut de Cardiologie, Hôpital Pitié-Salpêtrière (AP-HP), Paris 75013, France; Sorbonne Université, ACTION Study Group, INSERM UMRS 1166, Institut de Cardiologie, Hôpital Pitié-Salpêtrière (AP-HP), Paris 75013, France; Sorbonne Université, ACTION Study Group, INSERM UMRS 1166, Institut de Cardiologie, Hôpital Pitié-Salpêtrière (AP-HP), Paris 75013, France

**Keywords:** Cardiogenic shock, Acute myocardial infarction, Antiplatelet therapy, Pre-treatment, P2Y12 inhibitor, Bleeding

## Abstract

**Background and aims:**

There are currently no data regarding pre-treatment with P2Y12 inhibitors in patients with acute myocardial infarction complicated with cardiogenic shock (AMI-CS). This study investigates the effectiveness and safety of pre-treatment with P2Y12 inhibitors in patients with AMI-CS.

**Methods and results:**

Using the ACTION-SHOCK cohort, we included consecutive patients hospitalized between 2012 and 2023 with AMI-CS admitted for coronary angiography within 24 h of admission. Pretreatment was defined by the administration before angiography of an oral loading dose of a P2Y12 inhibitor. We evaluated the association between pretreatment and either major adverse cardiovascular events (MACE) or major bleeding at 30 days after admission, using an inverse probability weighting (IPW) approach. MACE was defined by the composite of all-cause death, ischaemic stroke, myocardial infarction, or stent thrombosis. Major bleeding was defined by Bleeding Academic Research Consortium grade 3, 4, or 5. Among the 421 patients with AMI-CS admitted to the catheterization laboratory within 24 h of admission, 224 (53.2%) patients received pre-treatment with a P2Y12 inhibitor. No association between pre-treatment with P2Y12 inhibitor and MACE at 30 days was found [42.1% vs. 38.8%—IPW hazard ratio (wHR): 1.11, 95% CI: 0.82–1.50]. Pre-treatment was associated with an increased risk of major bleeding (42.2% vs. 32.3%—wHR: 1.48, 95% CI: 1.05–2.08). The effect of pre-treatment on MACE or major bleeding at 30 days is consistent across STEMI/NSTEMI patients.

**Conclusion:**

In patients with AMI-CS, pretreatment with a P2Y12 inhibitor oral load was associated with an increased risk of major bleeding without benefit on MACE.

## Graphical Abstract

**Graphical Abstract:**
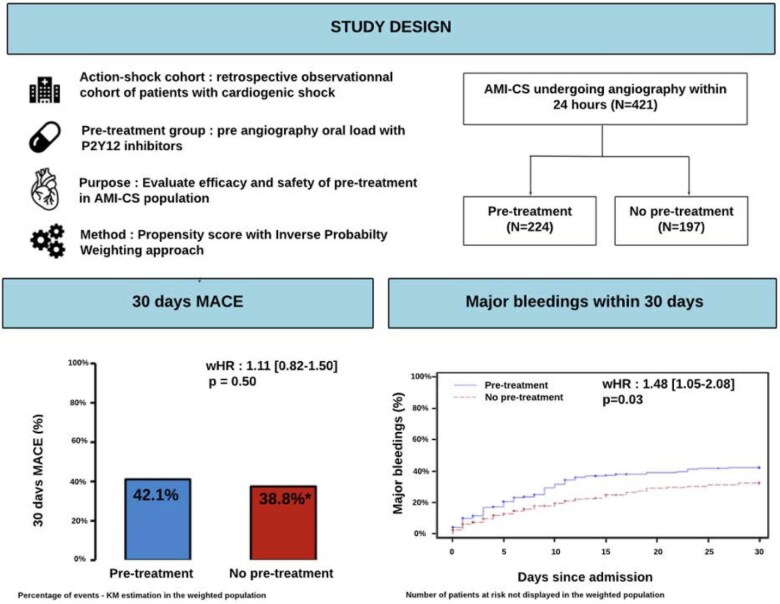

## Introduction

Cardiogenic shock (CS) is a serious complication of 48% of acute myocardial infarction (AMI) and remains an important cause of death with a rate of 30–45%.^1,2^ During AMI, an effective and safe antithrombotic therapy should antagonize the high platelet reactivity of CS,^3^ facilitate percutaneous coronary intervention (PCI), and reduce myocardial damage without increasing serious bleeding events. This need for efficacy and safety is often prevented by the low cardiac output, causing kidney failure, hepatic insufficiency, platelet dysfunction, and impaired enteric absorption.^4,5^ Subsequently, patients are exposed to major risk of ischaemic and haemorrhagic events, reaching 20% for stent thrombosis^6^ and 20–30% for bleeding in registries.^7,8^

While the question of pre-treatment with P2Y12 inhibitors has been well addressed in NSTEMI and STEMI, there are currently no data on patients with AMI-CS. Indeed, patients with CS were either excluded or underrepresented from previous studies focusing on pretreatment.^9–11^

Thus, our study aims to analyse the safety and effectiveness of a loading oral pretreatment dose of P2Y12 inhibitor in patients with suspected AMI-CS.

## Methods

### Study design and population

Using the ACTION-SHOCK cohort^12^ and the Pitié-Salpêtrière catheterization laboratory database (Paris, France), consecutive patients with AMI-CS admitted for coronary angiography between January 2012 and November 2023 were included in our study. Patients were retrospectively included until January 2022, then prospectively included.

The inclusion criteria were the presence before admission to the catheterization laboratory of

1. a verified cardiogenic shock defined by the presence of two of the following signs of CS: systolic blood pressure <90 mmHg (or mean blood pressure <65 mmHg) for >30 min or the use of dobutamine and/or noradrenalin to maintain a systolic pressure ≥90 mmHg or signs of pulmonary congestion (e.g. crackles oedema) or signs of impaired organ perfusion with at least one of the following criteria: altered mental status, cold and clammy skin and limbs, oliguria with a urine output <30 mL/h, or an arterial lactate level >2 mmol/L;
2. an AMI prompting coronary angiography within 24 h of admission. AMI was defined by a significant elevation of troponin (>2×N) associated with at least one of the following: chest pain or new ischaemic electrocardiogram changes or development of pathologic Q waves or new regional wall motion abnormality in a pattern consistent with an ischaemic aetiology.

Exclusion criteria were (i) coronary angiography performed more than 24 h of admission, (ii) initial presentation with cardiopulmonary arrest, (iii) shock from other causes (sepsis, anaphylaxis, tamponade, pulmonary embolism), (iv) haemorrhagic context such as a recent major surgery <1 month, (v) known allergy to P2Y12 inhibitors, and (vi) age <18 years or under guardianship.

### Ethical approvals

The protocol was approved by the ethics committee of Sorbonne University, Paris, France (CER-2022-047). An information letter was sent to each patient who matched the selection criteria stating their right to decline participation. In the absence of refusal, the patient was included in the study. Computer and paper reports were also checked for objections from deceased patients who were notified during their lifetime. If a third party had been designated as responsible for the execution of directives regarding the right to refuse the use of the deceased's data, an attempt was made to contact them to ensure nonopposition.

### Data collection and follow-up

The collected data were pre-specified in the research protocol of the ACTION-Shock registry. All data were collected by reviewing their electronic and paper medical records from either an emergency, medical service, catheterization laboratory, or intensive care unit (ICU). Endpoints were reviewed by two independent investigators. The prognostic score Society for Cardiovascular Angiography and Interventions (SCAI) SHOCK^13^ was used to evaluate CS severity within 24 h of admission. Each patient was followed-up during their hospital stay and up to 30 days.

### Definition of pre-treatment

Pre-treatment with P2Y12 inhibitor was defined as the administration of an oral loading dose of P2Y12 inhibitor before arriving to the catheterization laboratory. This loading dose was either 300 mg or 600 mg of clopidogrel, 60 mg of prasugrel, or 180 mg of ticagrelor.

### Angiographic procedure

In line with current practice, the radial artery was preferred for coronary angiography, with routine intra-arterial injection of low-molecular-weight heparin. Significant coronary artery disease was defined by the presence of at least one coronary lesion with ≥50% stenosis in the left main coronary artery or ≥70% stenosis in other major coronary vessels. A culprit lesion was identified by signs of plaque rupture, severe spasm, or coronary dissection. If a culprit coronary lesion was identified, primary PCI was performed. Revascularization of nonculprit lesions was performed in accordance with international guidelines. Coronary artery bypass grafting was considered necessary based on coronary anatomy and decided after collegial discussion between cardiologists and surgeons. Inotropes and mechanical assistance devices were used as required, depending on the patient's haemodynamic status. After the procedure, stable patients were admitted to the cardiology ICU, unstable patients and patients requiring mechanical support or invasive ventilation were admitted to the critical care unit.

### Antithrombotic regimen

The type, dosage, timing of initial administration, and management of antiplatelet therapy during the ICU stay were recorded for all patients. The antiplatelet treatment received was evaluated at three key time periods (i) upon admission to the catheterization laboratory, including any pre-hospital administration; (ii) during coronary angiography and PCI; and (iii) throughout the hospital stay. Patients in the no pre-treatment group who did not receive a P2Y12 inhibitor during PCI due to the inability to absorb oral medication were administered a loading dose after the PCI. The management of antithrombotic therapy was decided by the clinicians according to international guidelines.

### Outcomes and definitions

The primary effectiveness outcome was 30-day major adverse cardiovascular events (MACE), defined as the composite of all-cause death, myocardial infarction, definite or probable stent thrombosis, or ischaemic stroke.^14^ The primary safety outcome included major bleeding within 30 days. Secondary outcomes included each component of the primary composite effectiveness outcomes and the occurrence of acute peripheral ischaemia at 30 days.

Stent thrombosis was defined according to the Academic Research Consortium (ARC) criteria.^14^ Myocardial infarction was defined as type 1 MI according to the Fourth Universal Definition of Myocardial Infarction.^15^ Ischaemic stroke was defined by the presence of recent cerebral infarction documented on computed tomography or magnetic resonance imaging. Major bleeding was defined as BARC (Bleeding Academic Research Consortium) 3, 4, or 5 bleeding according to the BARC criteria.^16^

### Statistical analysis

Descriptive statistics were reported as median (interquartile range) for quantitative variables or *n* (%) for categorical variables. Quantitative variables were compared using a Wilcoxon test, and categorical variables were compared using a chi-squared test, or Fisher exact test when necessary. Time to outcomes (ischaemic events, bleeding, and type of bleeding) following hospitalization were analysed using the Kaplan-Meier method. Patients for which outcome was not observed were censored at the last date of contact or 30 days of follow-up.

To help account for the nonrandomized load of P2Y12 inhibitors, we use an inverse probability weighting approach to balance the differences in baseline variables between strategy groups. Patients’ probability of receiving a load of P2Y12 inhibitor (propensity score) was estimated using a multivariable logistic regression model given their baseline characteristics (age, sex, hypertension, diabetes, dyslipidaemia, current smoker, prior PCI, and ST elevation). Standardized mean differences were examined to assess balance, with a threshold of 10% indicating clinically meaningful imbalance (Supplementary material online, *Figure S1*).

The association between load of P2Y12 inhibitor and outcomes at 30 days (ischaemic events and bleeding) was evaluated with Cox model in the inverse probability weighted population and presented as weighted hazard ratio (wHR) with their 95% confidence interval.

To assess the consistency of the results in different subgroups (Electrocardiogram presentation, age, sex, and extracorporeal membrane oxygenation use), the interaction between subgroups and load of P2Y12 inhibitor was evaluated for MACE and major bleeding using Cox model in the inverse probability weighted population. All tests were two-sided with an α risk of 5%. No replacement of missing data was done. All analyses were performed using the SAS statistical software package, release 9.4 (SAS institute Inc., Cary, NC, USA) and R Statistical Software version 4.4.1.

## Results

### Patients

**Figure 1:**
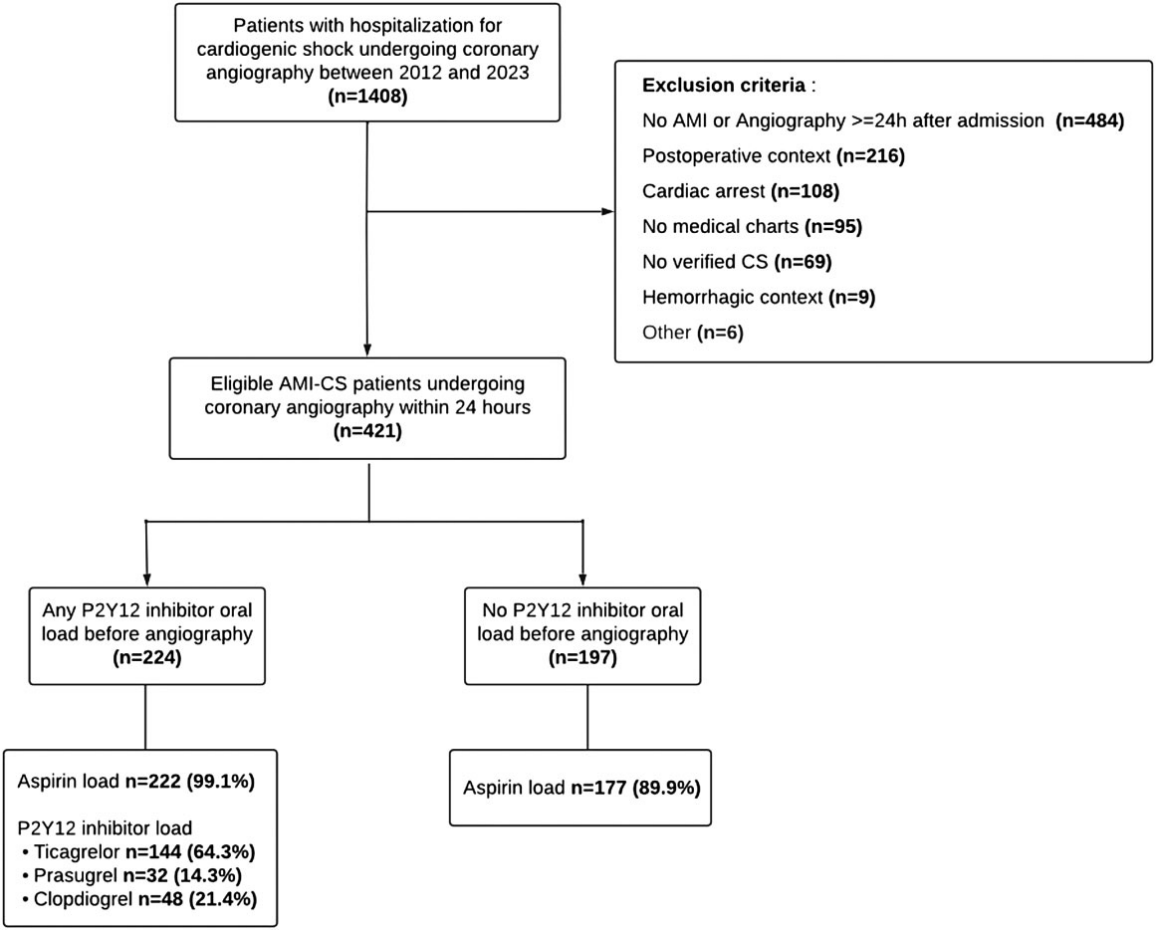
Flow chart. AMI, acute myocardial infarction; AMI-CS, acute myocardial infarction with cardiogenic shock; CS, cardiogenic shock.

Between January 2012 and November 2023, 421 patients were admitted to the catheterization laboratory within 24 h of an ongoing AMI-CS, of whom 224 (53.2%) received a loading dose of a P2Y12 inhibitor before. Among the 224 patients with pre-treatment, 144 (64.3%) received a loading dose of ticagrelor, 32 (14.3%) of prasugrel, and 48 (21.4%) of clopidogrel. Out of the 197 patients without pre-treatment, 177 (89.8%) received an intravenous or oral loading dose of aspirin (*Figure 1*).

In the without pre-treatment group, patients were more frequently treated with daily anticoagulation (12.2% vs. 5.4% of the patients with pre-treatment), had more frequently a PCI history (31.5% vs. 16.1% of the patients with pre-treatment) and more frequently a peripheral artery disease history (12.7% vs. 5.8% of the pre-treated patients) compared with patients with pre-treatment. Patients with pre-treatment more frequently felt chest pain (89.7% vs. 76.1%) and presented more often with ST elevation myocardial infarction (87.9% vs. 60.4%) (*Table  1* and Supplementary material online, *Tables S1* and *S2*).

**Table 1:** Baseline characteristics according to the pre-treatment strategy

	Pre-treatment with P2Y12 inhibitor	No pre-treatment with P2Y12 inhibitor
	(*N* = 224)	(*N* = 197)
Demographics		
Age (years)	66.4 [58.3–77.3]	69.0 [57.4–77.1]
Female sex	68 (30.4%)	60 (30.5%)
Body mass index (kg/m²)	25.4 [23.0–27.9]	24.6 [22.4–27.7]
Cardiovascular risk factors		
Hypertension	125 (55.8%)	111 (56.3%)
Type 2 diabetes	70 (31.3%)	71 (36.0%)
Dyslipidaemia	91 (40.6%)	95 (48.2%)
Current smoking	92 (41.1%)	86 (43.7%)
Cardiovascular history		
Prior PCI	36 (16.1%)	62 (31.5%)
Prior CABG	7 (3.1%)	7 (3.6%)
Prior stroke	15 (6.7%)	14 (7.1%)
Prior PAD	13 (5.8%)	25 (12.7%)
Prior hospitalization for HF or CS	14 (6.3%)	27 (13.7%)
Atrial fibrillation	10 (4.5%)	21 (10.7%)
Current medication before admission		
Aspirin	57 (25.4%)	72 (36.5%)
Clopidogrel	21 (9.4%)	25 (12.7%)
Prasugrel	0 (0.0%)	3 (1.5%)
Ticagrelor	1 (0.4%)	3 (1.5%)
Oral anticoagulation	12 (5.4%)	24 (12.2%)
Clinical presentation		
Chest pain	201 (89.7%)	150 (76.1%)
ST elevation	197 (87.9%)	119 (60.4%)
MBP (mmHg)	63.3 [56.2–67.8]	64.7 [59.8–70.0]
Heart rate (BPM)	100.0 [85.0–115.0]	105.5 [86.0–120.0]
SCAI		
A/B	41 (18.3%)	29 (14.7%)
C	107 (47.8%)	108 (54.8%)
D	32 (14.3%)	28 (14.2%)
E	44 (19.6%)	32 (16.2%)
Echocardiographic data		
LVEF (%)	30.0 [20.0–40.0]	25.0 [20.0–35.0]
Mitral regurgitation (moderate or severe)	20/216 (9.3%)	28/193 (14.5%)
Mechanic complication	13/217 (6.0%)	9/193 (4.7%)

BPM, beats per minute; CABG, coronary artery bypass grafting; CS, cardiogenic shock; HF, heart failure; LVEF, left ventricular ejection fraction; MBP, mean blood pressure; PAD, peripheral artery disease; PCI, percutaneous coronary intervention.

### Procedural characteristics and angiographic findings

Coronary angiography was performed with a median time after the onset of symptoms of 7 [5; 30] h and 14 [5; 30] h, respectively. The radial artery was the main vascular access for coronarography procedure (80.9%). Patients who did not receive pre-treatment were more likely to have no significant coronary disease identified (10.7% vs. 4.5% in the pre-treatment group). PCI was more frequently performed in the pre-treatment group [186 (83.0%) patients vs. 130 (66.0%) patients in the no pre-treatment group] (*Table  2*).

**Table 2:** Procedural characteristics according to the pre-treatment strategy

	Pre-treatment with P2Y12 inhibitor	No pre-treatment with P2Y12 inhibitor
	(*N* = 224)	(*N* = 197)
Immediate procedure	186 (83.0%)	130 (66.0%)
Time from first symptom to angiography (hour)	7.0 [4.0–19.0]	14.0 [5.0–30.0]
Procedure access		
Radial	154/192 (80.2%)	146/179 (81.6%)
Femoral	36/192 (18.8%)	30/179 (16.8%)
Other	2/192 (1.0%)	3/179 (1.7%)
Initial TIMI		
0	149 (66.5%)	92 (46.7%)
1	16 (7.1%)	25 (12.7%)
2	25 (11.2%)	29 (14.7%)
3	34 (15.2%)	51 (25.9%)
Final TIMI		
0	34 (15.2%)	28 (14.2%)
1	9 (4.0%)	17 (8.6%)
2	33 (14.7%)	20 (10.2%)
3	148 (66.1%)	132 (67.0%)
No significant disease	10 (4.5%)	21 (10.7%)
Number of vessels with lesion		
0	10 (4.5%)	21 (10.7%)
1	55 (24.5%)	50 (25.3%)
2	82 (36.6%)	47 (23.9%)
3	77 (34.4%)	79 (40.1%)
PCI revascularization	186 (83.0%)	130 (66.0%)
Stenting		
LMCA	18 (8.0%)	16 (8.1%)
LAD	101 (45.1%)	60 (30.5%)
LCX	32 (14.3%)	34 (17.3%)
RCA	47 (21.0%)	37 (18.8%)
CABG revascularization	15 (6.7%)	14 (7.1%)
ICU management^a^		
Dobutamine use	199/218 (91.3%)	180/194 (92.8%)
ECMO	59/218 (27.1%)	45/194 (23.2%)
IABP	40/218 (18.3%)	32/194 (16.5%)
Invasive ventilation	86/218 (39.4%)	79/194 (40.7%)
Lactates (mmol/L)^b^	3.3 [2.2–5.0]	3.1 [2.1–4.8]

CABG, coronary artery bypass grafting; ECMO, extracorporeal membrane oxygenation; IABP, intra-aortic balloon pump; ICU, intensive care unit; LAD, left anterior descending artery; LCX, left circumflex artery; LMCA, left main coronary artery; PCI, percutaneous coronary intervention; RCA, right coronary artery.

a Nine patients deceased in catheterization laboratory before ICU admission were excluded: six in the pre-treatment group and three in the no pre-treatment group.

b Missing data: 33 for pre-treatment group and 22 no pre-treatment group.

### Antithrombotic management in the catheterization laboratory and during hospitalization

Among patients without pre-treatment, 94 (47.7%) received a loading dose of an oral P2Y12 inhibitor in the catheterization laboratory, 26 (13.2%) received cangrelor and 46 (23.4%) received GPI inhibitors. Overall, 348 patients (84.5%) received at least one dose of a P2Y12 inhibitor orally during hospitalization (199, 91.3% for pre-treatment group and 149, 76.8% for the no pre-treatment group). There is an overall decrease in the pre-treatment administration over the years (63.9% in 2014 vs. 48.0% in 2023), with a relative increase in ticagrelor use (40.0% in 2014 vs. 95.8% in 2023) and a reduction in prasugrel and clopidogrel utilization. Detailed antithrombotic management data are available in *Table  3*, Supplementary material online, *Table  S3* and *Figure S2*.

**Table 3:** Antithrombotic management according to the pre-treatment strategy

	Pre-treatment with P2Y12 inhibitor	No pre-treatment with P2Y12 inhibitor
	(*N* = 224)	(*N* = 197)
Per angiography management		
P2Y12 inhibitor load^a^	19 (8.5%)	94 (47.7%)
Ticagrelor	11 (4.9%)	43 (21.8%)
Prasugrel	6 (2.7%)	27 (13.7%)
Clopidogrel	2 (0.9%)	24 (12.2%)
Cangrelor use	6 (2.7%)	26 (13.2%)
GPI inhibitor use	70 (31.3%)	46 (23.4%)
During hospitalization^b,c^		
Aspirin use	211/218 (96.8%)	179/194 (92.3%)
P2Y12 inhibitor use	199/218 (91.3%)	149/194 (76.8%)
Ticagrelor	134/218 (61.5%)	76/194 (39.2%)
Prasugrel	43/218 (19.7%)	40/194 (20.6%)
Clopidogrel	66/218 (30.3%)	63/194 (32.5%)
Oral anticoagulation (VKA or DOACs)	26/218 (11.9%)	30/194 (15.5%)

DOAC, direct oral anticoagulants; VKA, vitamin K antagonist.

a Patients from pre-treatment group could have received another loading dose of P2Y12 inhibitor during angiography.

b Nine patients deceased in catheterization laboratory before ICU admission: six in the pre-treatment group and three in the no pre-treatment group.

c Use was defined as at least one standard daily dose administered (75 mg of aspirin, 75 mg of clopidogrel, 10 mg of prasugrel, 90 mg*2 of ticagrelor).

### Effectiveness of pre-treatment

**Figure 2:**
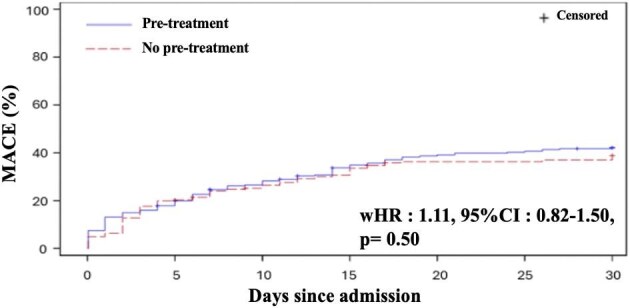
Kaplan Meier estimates of the primary outcome. IPW analysis. Kaplan Meier estimates of (all cause death, MI, ischaemic stroke, or stent thrombosis) at 30 days according to the pre-treatment strategy groups.

No association between pre-treatment by P2Y12 inhibitors and occurrence of MACE at 30 days was found (wHR: 1.11, 95% CI: 0.82–1.50, *Figure 2* and *Table  4*). Similarly, there was no association between pre-treatment by P2Y12 inhibitors and occurrence of all-cause death at 30 days (wHR: 1.11, 95% CI: 0.80–1.55, *Table  4*).

**Table 4:** Primary and secondary effectiveness outcomes at 30 days–IPW analysis

	Pre-treatment with P2Y12 inhibitor	No pre-treatment with P2Y12 inhibitor	
Outcome	(*N* = 224)	(*N* = 197)	wHR^a^ (95%CI) (*P*-value)
MACE at 30 days^b^	90 **42.1%** [35.4–49.6]	73**38.8%** [32.0–46.4]	**1.11 [0.82–1.50] (0.50)**
Death from any cause at 30 days^b^	7935.7% [29.2–43.2]	6232.1% [25.7–39.6]	1.11 [0.80–1.55] (0.53)
Ischaemic stroke at 30 days^b^	147.8% [4.6–13.3]	129.0% [5.3–15.1]	0.92 [0.45–1.89] (0.81)
Peripheric ischaemia at 30 days^b^	116.2% [3.3–11.4]	95.5% [2.8–10.9]	1.19 [0.50–2.85] (0.70)
New MI at 30 days^b^	84.6% [2.1–9.9]	21.6% [0.4–6.1]	3.03 [0.72–12.80] (0.13)
Stent thrombosis definite or probable at 30 days^b,c^	137.2% [4.1–12.6]	53.4% [1.4–8.1]	2.16 [0.82–5.70] (0.12)

a Inverse probability weighted Cox proportional hazard model. Variables included in the propensity score: age, sex, hypertension, diabetes, dyslipidaemia, current smoker, prior PCI, and ST elevation.

b Number of events—Kaplan Meier estimate [95% CI].

c Pre-treatment group: four definite ≤24 h stent thrombosis, six definite stent thrombosis between 24 h and 30 days and three probable stent thrombosis. No pre-treatment group: two definite ≤24 h stent thrombosis, two definite stent thrombosis between 24 h and 30 days and one probable stent thrombosis.

### Safety of pre-treatment

**Figure 3:**
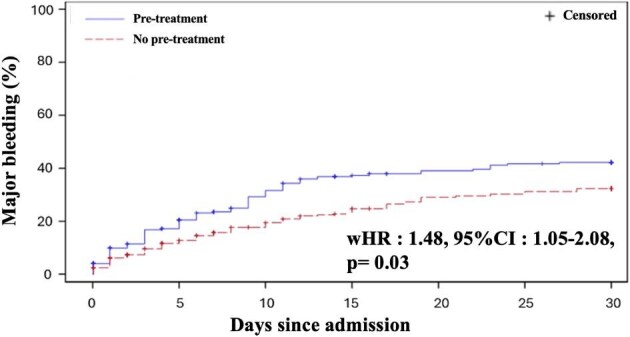
Kaplan Meier estimates of the safety outcome. IPW analysis. Kaplan Meier estimates of (BARC 3, 4, or 5) bleeding at 30 days according to the pre-treatment strategy groups.

Major bleeding within 30 days occurred for 80 patients (42.2%) patients of the pretreatment group and 48 (32.3%) patients of the no-pretreatment group (*Figure 3*). The occurrence of major bleeding at 30 days was significantly higher in the pre-treatment group compared to the no pre-treatment group (wHR: 1.48, 95% CI: 1.05–2.08). Bleeding outcomes are summarized in *Table  5*. The most frequent bleeding complications involved gastrointestinal, urologic, and femoral bleeding. All bleeding locations are shown in Supplementary material online, *Table  S4*.

**Table 5:** Safety outcomes at 30 days–IPW analysis

	Pre-treatment with P2Y12 inhibitor	No pre-treatment with P2Y12 inhibitor	
Outcome	(*N* = 224)	(*N* = 197)	wHR^a^ (95%CI) (*P*-value)
Major bleeding (BARC3/4/5) at 30 days^b^	80**42.2%** [34.8–50.4]	48**32.3%** [25.3–40.6]	**1.48 [1.05; 2.08] (0.03)**
Number of patients with BARC2 bleeding within 30 days	27	23	
Number of patients with BARC3 bleeding within 30 days	69	41	
Number of patients with BARC4 (CABG related) bleeding within 30 days	4	2	
Number of patients with BARC5 bleeding within 30 days	7	6	

a Inverse probability weighted Cox proportional hazard model. Variables included in the propensity score: age, sex, hypertension, diabetes, dyslipidaemia, current smoker, prior PCI, and ST elevation.

b Number of events—Kaplan Meier estimate (95% CI).

### Subgroup analysis

**Figure 4:**
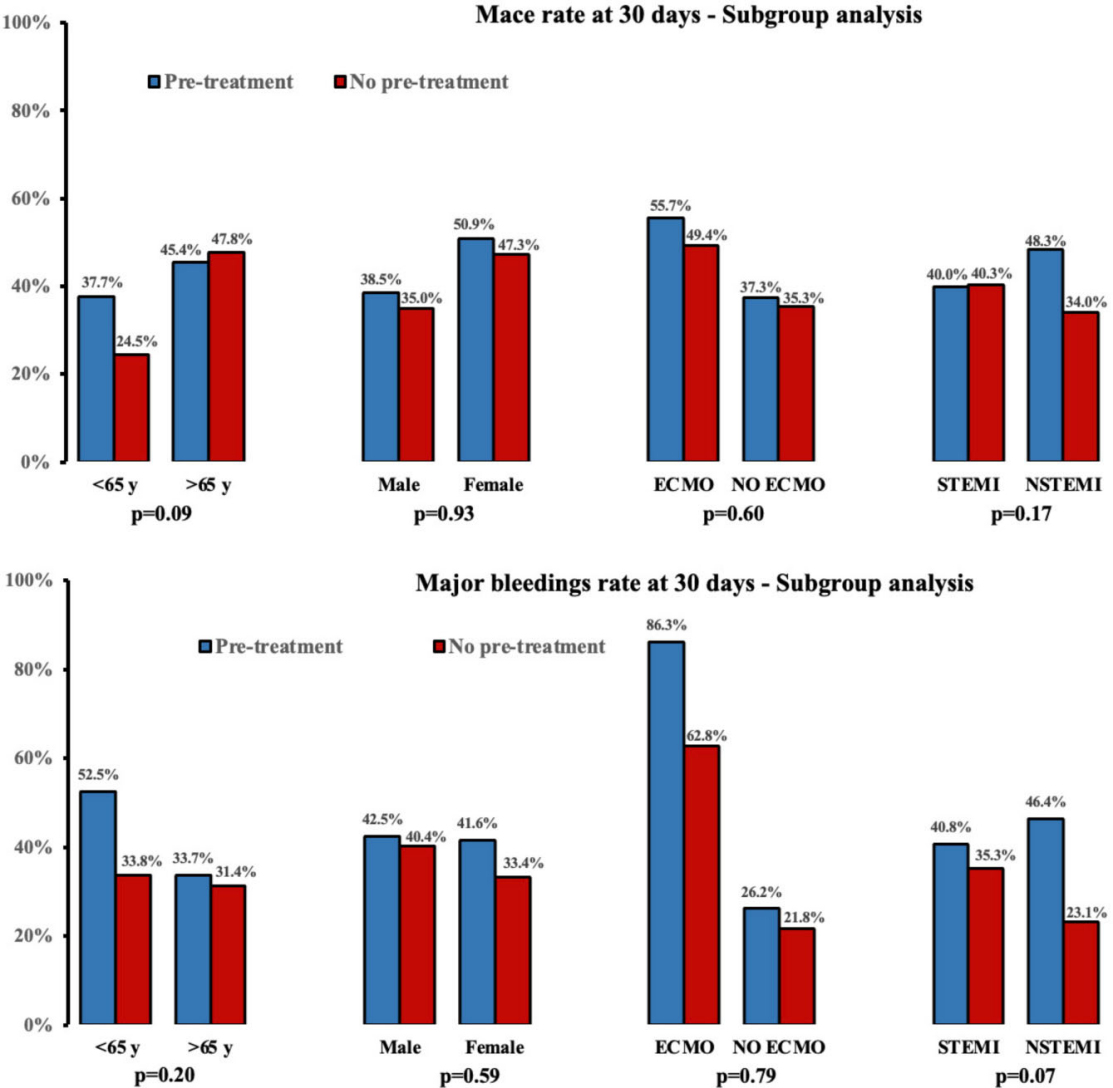
Subgroup analysis. Central illustration: Pre-treatment with P2Y12 inhibitor in acute myocardial infarction with cardiogenic shock.

Among the 316 patients in the STEMI group, MACE at 30 days occurred in 77 (40.0%) patients who received pre-treatment and 47 (40.3%) of those who did not. In this subgroup, the occurrence of major bleeding was 68 (40.8%) in the pre-treatment group and 34 (35.3%) in the no pre-treatment group. The effect of pre-treatment on MACE or major bleeding at 30 days is consistent across STEMI/NSTEMI patients and across other subgroups (*Figure 4*). Detailed characteristics and outcomes in the STEMI population are displayed in Tables S5–S8.

## Discussion

In this study of 421 patients with AMI-CS evaluating the effectiveness and safety of pre-treatment with P2Y12 inhibitors, the main findings were as follows: firstly, pre-treatment with P2Y12 inhibitor was administered in half of the patients with AMI-CS before reaching the catheterization laboratory; secondly, absence of pre-treatment with oral P2Y12 inhibitor was not associated with a higher risk of MACE; thirdly, pre-treatment with P2Y12 inhibitor was associated with a greater risk of major bleeding complications at 30 days. Eventually, the effect of pre-treatment on MACE or major bleeding at 30 days is consistent across STEMI/NSTEMI patients.

No association was found between pre-treatment and MACE occurrence, consistent with the ACCOAST trial results that included patients with NSTEMI without cardiogenic shock.^9^ This absence of benefit from P2Y12 inhibitors pre-treatment remains debated in STEMI patients.^17^ Indeed, while the ATLANTIC trial was not conclusive in a population of STEMI patients without cardiogenic shock,^10^ the ATLANTIC H-24 subanalysis showed a signal about a reduced rate of ischaemic events at 24 h and a reduced rate of stent thrombosis at 30 days with pre-treatment.^18^ More recently, the CREA ARIAM Andalucía registry reported a significant reduction in MACE with P2Y12 inhibitor pre-treatment. However, no benefit was shown in the subgroup analysis concerning the 6.7% of patients with CS included.^19^ However, we did not demonstrate a reduction in ischaemic events with pre-treatment in patients with AMI-CS.

Several factors may explain the absence of the effect of pre-treatment in our shock population. Firstly, the antiplatelet effects of oral P2Y12 inhibitors are most probably delayed in AMI-CS patients due to slower absorption, altered metabolism, and concomitant therapies.^3,5,20^ This delay may be too long to prevent acute thrombotic complications in the catheterization laboratory but could not avoid bleeding complications occurring often later in the ICU. Secondly, oral pre-treatment with P2Y12 inhibitors may be futile in patients with cardiogenic shock due to the severity of their prognosis. Thirdly, the absence of pre-treatment was often counterbalanced by a higher use of intravenous cangrelor therapy, 2.7% in patients of the pre-treated group vs. 13.2% in patients without pre-treatment. As suggested by Droppa *et al.*,^21^ with a potent and rapid effect, intravenous antithrombotic such as cangrelor may be more effective than oral P2Y12 inhibitors in AMI-CS patients.

Patients who received P2Y12 inhibitors before arriving at the catheterization laboratory were at higher risk of major bleeding compared with patients who did not receive pre-treatment. With respective rates of 42.2% and 32.3%, bleeding rates at 30 days were higher in this study compared to other studies, 21.5% in the CULPRIT SHOCK subanalysis,^7^ 16.5% from ECLS-SHOCK,^22^ and around 20% from DanGer-SHOCK.^23^ This can be explained by the clinical severity of the patients in this cohort (83.5% of patients were classified as SCAI C, D, or E), 25.2% of patients received extracorporeal membrane oxygenation support, 40.0% invasive ventilation, 24.6% renal replacement therapy, and 6.9% required surgical revascularization. This number of patients who underwent coronary artery bypass graft may also explain the higher occurrence of major bleeding complications with pre-treatment. Indeed, pre-treatment by P2Y12 inhibitors was shown to increase bleeding and mortality in patients receiving surgical revascularization.^24^

Currently, no trial specifically addressed P2Y12 inhibitors pre-treatment in AMI-CS patients, and the 2021 ESC position paper about antithrombotic management in AMI-CS did not provide any recommendation regarding pre-treatment due to lack of data.^25^ This absence of consensus in AMI-CS is well reflected in this study, where nearly as many patients received pre-treatment with a P2Y12 inhibitor (53.2%) as did not receive pre-treatment. The ongoing trial DAPT SHOCK MI (Dual Antiplatelet Therapy for Shock Patients with Acute Myocardial Infarction; NCT03551964), a multicentre, double-blind trial that randomizes at the catheterization laboratory the P2Y12 receptor blockade with intravenous cangrelor vs. ticagrelor in ∼550 AMI-CS patients, will soon provide important data on antithrombotic management in this population. Pending further randomized trials, our results support a strategy of delayed or more selective administration of P2Y12 inhibitor until coronary anatomy is known and PCI is confirmed as the treatment strategy, extending the results of the ACCOAST trial to this population of cardiogenic shock.^9^

We acknowledge several limitations in our study. Firstly, potential cofounding factors and other biases may have persisted despite inverse propensity weighting. Secondly, this is a single tertiary centre study reflecting the current practices of transferring centres and emergency services in our wide geographic area. Thirdly, the distribution of patients presenting with STEMI was not balanced between the groups, although we cannot exclude that the increased risk of major bleeding associated with pre-treatment could be reduced in this subgroup. Nevertheless, this limitation was addressed by weighting the population based on several criteria, including the ST-segment presentation. An important limitation is the difficulty of attributing the higher bleeding rate observed in the pre-treatment group exclusively to the administration of pre-treatment. This group underwent coronary angioplasty more frequently and had higher use of P2Y12 inhibitors during hospitalization. Therefore, the overall exposure to antithrombotic therapy may have contributed to the higher bleeding rate observed in the pre-treatment group. The absence of data regarding target temperature management, proton pump inhibitors, and opioid use is also a limitation, as these are known factors that may influence the pharmacology of P2Y12 inhibitors. Further studies should consider including these variables to better understand their interaction with pre-treatment. Additionally, it is known that there is a relationship between the timing of pre-treatment administration and the antithrombotic efficacity of MACE. However, due to the absence of data, we were unable to explore the influence of the timing administration in this study.

## Conclusion

Among patients with AMI complicated by cardiogenic shock, pre-treatment with an oral P2Y12 inhibitor does not appear to be associated with lower rates of ischaemic outcomes but was associated with a higher risk of major bleeding complications. These results suggest that the administration of P2Y12 inhibitors could be delayed until the results of the coronary angiography and if PCI is selected as the revascularization strategy.

## Supplementary Material

pvaf019_Supplemental_File

## Notes

**Conflict of interest**: A.C. reports research funds from Getinge and lecture fees from Bayer, Baxter, Getinge, and Xenios-Fresenius. M.K. reports research funds for the institution from ACTION-Coeur, PHRC, Eligo, and Fédération Française de Cardiologie, consulting fees from Eligo, Kiniksa, Sanofi and fees from Bayer, Sanofi, and Servier. J.S. reports fees or a contribution to transport costs from AstraZeneca, Bayer HealthCare SAS, Abbott Medical France SAS, Biotronik, Boehringer Ingelheim France, CSL Behring SA, Gilead Science, sanofi-aventis France, Stockholder of Pharmaseeds, Terumo France SAS, and Zoll. G.M. reports research funds for the Institution or consulting/lecture fees from Abbott, Amgen, AstraZeneca, Bayer, BMS, Boehringer-Ingelheim, Celecor, CSL Behring, Hexacath, Idorsia, Lilly, Novo Nordisk, Pfizer, SMT, and Terumo. M.Z. reports research grants from Bayer, BMS Pfizer, the Fédération Française de Cardiologie, and Servier. All other authors have reported that they have no relationships relevant to the contents of this paper to disclose.

## Data availability

The data underlying this article will be shared on reasonable request to the corresponding author.
