# Supplementary material for: Pre-treatment with P2Y12 inhibitors in acute myocardial infarction with cardiogenic shock
Source: Eur Heart J Cardiovasc Pharmacother. 2025 Apr 1;11(6):489–99. doi: 10.1093/ehjcvp/pvaf019 (PMC12450598; doi:10.1093/ehjcvp/pvaf019)
Supplement: pvaf019_Supplemental_File [file pvaf019_supplemental_file.docx]

**SUPPLEMENTAL MATERIAL**

**Table S1. Intensive Care Unit management characteristics**

**Table S2. Biological data**

**Table S3. Yearly description of pre-treatment agent**

**Table S4. Major bleeding description.**

**Table S5. Baseline characteristics according to the pre-treatment strategy – STEMI population**

**Table S6. Procedural characteristics according to pre-treatment strategy – STEMI population**

**Table S7. Antithrombotic Management according to pre-treatment strategy – STEMI population.**

**Table S8. Outcomes at 30 days in the STEMI population – IPW analysis.**

**Figure S1. Standardized mean differences for variables included in the propensity score model**

**Figure S2. Yearly Distribution of Pre-Treatment**

**Table S1. Intensive Care Unit management characteristics**

|  | **Pre-treatment with P2Y12 inhibitor** | **No pre-treatment with P2Y12 inhibitor** |
| --- | --- | --- |
|  | **(N=218^a^)** | **(N=194^a^)** |
| **Amine** |  |  |
| Dobutamine use | 199 (91.3%) | 180 (92.8%) |
| Noradrénaline use | 129 (59.2%) | 110 (56.7%) |
| Number of days of amines^b^ | 4.0 [2.0-8.0] | 4.0 [3.0-8.0] |
| **Support** |  |  |
| ECMO | 59 (27.1%) | 45 (23.2%) |
| IABP | 40 (18.3%) | 32 (16.5%) |
| Assist duration (days)^c^ | 7.0 [3.0-11.0] | 6.5 [3.5-15.0] |
| Invasive ventilation | 86 (39.4%) | 79 (40.7%) |
| Renal replacement therapy | 59 (27.1%) | 42/190 (21.9%) |
| **Transplantation during hospit** | 3 (1.4%) | 3 (1.5%) |
| **Cardioprotect introduction** |  |  |
| Betablocker | 105 (48.2%) | 85 (43.8%) |
| ACEi | 136 (62.4%) | 125 (64.4%) |
| Aldosterone blocker | 60 (27.5%) | 51 (26.3%) |
| SGLT2 inhibitor | 15 (6.9%) | 14 (7.2%) |
| **Hospitalization duration (days)^d^** | 12.0 [7.0-21.0] | 13.0 [6.0-25.0] |

^a^ 9 patients deceased in catheterization laboratory before ICU admission: 6 in the pretreatment group and 3 in the no pretreatment group

^b^ in patients with Dobutamine and/or Noradrenaline use

^c^ in patients with ECMO and/or IABP

^d^ In total population : 224 for pre-treatment group and 197 for no pre-treatment group.

ACEi: Angiotensin-Converting Enzyme Inhibitor; ECMO: Extracorporeal Membrane Oxygenation; IABP: Intra Aortic Balloon Pump; SGLT2: Sodium-Glucose Cotransporter 2

**Table S2. Biological data**

|  | **Pre-treatment with P2Y12 inhibitor** | | **No pre-treatment with P2Y12 inhibitor** |
| --- | --- | --- | --- |
|  | **(N=224)** | | **(N=197)** |
| **Hemoglobin (g/dl) ^a^** | 12.9 [11.5-14.3] | 12.5 [11.0-13.7] | |
| **Platelets (x10^9/L) ^a^** | 231.0 [176.5-296.0] | 249.0 [179.0-325.5] | |
| **Creatinine (umol/l)** |  |  | |
| First^b^ | 103.0 [78.0-136.0] | 110.5 [83.0-149.0] | |
| Pic^c^ | 128.0 [97.0-186.0] | 144.0 [103.0-219.0] | |
| **NT pro BNP (ng/L)^a^** | 4281 [1091.0-8340.0] | 6606.5 [2154.5-17486.0] | |
| **Troponin (ng/L)** |  |  | |
| First^b^ | 1500 [293.0-5284.0] | 1184.0 [246.0-3745.0] | |
| Pic ^c^ | 7599.0 [3163.0-17152.0] | 3719.0 [1150.0-10550.0] | |
| xND ^d^ | 502.0 [155.0-1032.0] | 199.0 [58.0-714.0] | |
| **PH *** | 7.4 [7.3-7.4] | 7.4 [7.3-7.4] | |

^a^ Worst value during the first 24 hours after admission.

^b^ First value available value.

^c^ Highest value during hospitalization.

^d^ Normal value for troponin was 14 ng/L

**Table S3. Yearly description of pre-treatment agent**

| **Years** | **Pre-treatment with P2Y12 inhibitor** | | | **Total** |
| --- | --- | --- | --- | --- |
|  | **Ticagrelor (n=144)** | **Prasugrel (n=32)** | **Clopidogrel (n=48)** | **n = 224/421** |
| 2012 | 0/13 (0.0%) | 7/13 (53.8%) | 6/13 (46.2%) | 13/25 (52.0%) |
| 2013 | 2/13 (15.4%) | 4/13 (30.8%) | 7/13 (53.8%) | 13/21 (61.9%) |
| 2014 | 7/23 (30.0%) | 9/23 (40.0%) | 7/23 (30.0%) | 23/36 (63.9%) |
| 2015 | 7/21 (33.3%) | 7/21 (33.3%) | 7/21 (33.3%) | 21/45 (46.7%) |
| 2016 | 21/28 (75.0%) | 1/28 (3.6%) | 6/28 (21.4%) | 28/47 (59.6%) |
| 2017 | 18/24 (75.0%) | 3/24 (12.5%) | 3/24 (12.5%) | 24/41 (58.6%) |
| 2018 | 12/13 (92.3%) | 0/13 (0.0%) | 1/13 (7.7%) | 13/29 (44.8%) |
| 2019 | 18/24 (75.0%) | 1/24 (4.2%) | 5/24 (20.8% | 24/44 (54.5%) |
| 2020 | 16/19 (84.2%) | 0/19 (0.0%) | 3/19 (15.8%) | 19/37 (51.3%) |
| 2021 | 8/10 (80.0%) | 0/10 (0.0%) | 2/10 (20.0%) | 10/22 (45.5%) |
| 2022 | 12/12 (100.0%) | 0/12 (0.0%) | 0/12 (0.0%) | 12/24 (50.0%) |
| 2023 | 23/24 (95.8%) | 0/24 (0.0%) | 1/24 (4.2%) | 24/50 (48.0%) |

**Table S4. Major bleeding site description**

| **Bleeding site** | **Pre-treatment with P2Y12 inhibitor** | **No pre-treatment with P2Y12 inhibitor** |
| --- | --- | --- |
|  | **(80 bleeding)** | **(48 bleeding)** |
| Cerebral bleeding | 4 | 3 |
| Digestive bleeding | 25 | 14 |
| Thoracic bleeding | 18 | 9 |
| Femoral bleeding | 19 | 16 |
| ENT bleeding | 4 | 5 |
| Haematuria | 19 | 10 |
| Other bleeding (classified in radial or other) | 4 | 8 |
| Unidentified bleeding | 9 | 5 |
| ECMO related bleeding | 15 | 12 |
| ENT: Ear, Nose and Throat. (otorhinolaryngology bleeding) | | |

**Table S5. Baseline characteristics according to the pre-treatment strategy – STEMI population**

|  | **Pre-treatment with P2Y12 inhibitor** | **No pre-treatment with P2Y12 inhibitor** |
| --- | --- | --- |
|  | **(N=197)** | **(N=119)** |
| **Demographics** |  |  |
| Age (years) | 66.3 [57.7-77.1] | 68.5 [57.3 - 78.6] |
| Female sex | 61 (31.0%) | 35 (29.4%) |
| Body mass index (kg/m²) | 25.4 [23.3-27.8] | 25.2 [22.1-28.5] |
| **Cardiovascular risk factors** |  |  |
| Hypertension | 102 (51.8%) | 59 (49.6%) |
| Type 2 diabetes | 60 (30.5%) | 41 (34.5%) |
| Dyslipidemia | 69 (35.0%) | 52 (43.7%) |
| Current smoking | 81 (41.1%) | 55 (46.2%) |
| **Cardiovascular history** |  |  |
| Prior PCI | 25 (12.7%) | 31 (26.1%) |
| Prior CABG | 3 (1.5%) | 2 (1.7%) |
| Prior stroke | 11 (5.6%) | 11 (9.2%) |
| Prior PAD | 10 (5.1%) | 9 (7.6%) |
| Prior hospitalization for HF or CS | 7 (3.6%) | 11 (9.2%) |
| Atrial fibrillation | 6 (3.0%) | 11 (9.2%) |
| **Current medication before admission** |  |  |
| Aspirin | 42 (21.3%) | 42 (35.3%) |
| Clopidogrel | 14 (7.1%) | 12 (10.1%) |
| Prasugrel | 0 (0.0%) | 2 (1.7%) |
| Ticagrelor | 0 (0.0%) | 3 (2.5%) |
| Oral anticoagulation | 9 (4.6%) | 12 (10.1%) |
| **Clinical presentation** |  |  |
| Chest pain | 179 (90.9%) | 104 (87.4%) |
| MBP (mmHg) | 63.3 [56.7-68.0] | 63.3 [58.3-69.3] |
| Heart Rate (BPM) | 100.0 [82.0-113.0] | 110.0 [90.0-120.0] |
| SCAI |  |  |
| A/B | 33 (16.8%) | 10 (8.4%) |
| C | 96 (48.7%) | 68 (57.1%) |
| D | 29 (14.7%) | 16 (13.4%) |
| E | 39 (19.8%) | 25 (21.0%) |
| **Echocardiographic data** |  |  |
| LVEF (%) | 30.0 [20.0-40.0] | 30.0 [20.0-35.0] |
| Mitral regurgitation (moderate or severe) | 17/189 (9.0%) | 8/116 (6.9%) |
| Mechanic complication | 11/190 (5.8%) | 8/116 (6.9%) |

BPM: Beats per minute; CABG: coronary artery bypass grafting; CS: cardiogenic shock; HF: heart failure; LVEF: left ventricular ejection fraction; MBP: mean blood pressure; PAD: peripheral artery disease; PCI: percutaneous coronary intervention

**Table S6. Procedural characteristics according to the pre-treatment strategy – STEMI population**

|  | **Pre-treatment with P2Y12 inhibitor** | **No pre-treatment with P2Y12 inhibitor** |
| --- | --- | --- |
|  | **(N=197)** | **(N=119)** |
| **Immediat procedure** | 174 (88.3%) | 97 (81.5%) |
| **Time from first symptom to angiography (hour)** | 6.0 [4.0-18.0] | 9.0 [4.0-26.0] |
| **Procedure access** |  |  |
| Radial | 138/168 (82.1%) | 86/108 (79.6%) |
| Femoral | 28/168 (16.7%) | 20/108 (18.5%) |
| Other | 2/168 (1.2%) | 2/108 (1.9%) |
| **Initial TIMI** |  |  |
| 0 | 141 (71.6%) | 74 (62.2%) |
| 1 | 15 (7.6%) | 15 (12.6%) |
| 2 | 18 (9.1%) | 16 (13.4%) |
| 3 | 23 (11.7%) | 14 (11.8%) |
| **Final TIMI** |  |  |
| 0 | 30 (15.2%) | 15 (12.6%) |
| 1 | 9 (4.6%) | 10 (8.4%) |
| 2 | 31 (15.7%) | 14 (11.8%) |
| 3 | 127 (64.5%) | 80 (67.2%) |
| **No significant disease** | 10 (4.5%) | 21 (10.7%) |
| **Number of vessels with lesion** |  |  |
| 0 | 8 (4.1%) | 6 (5.0%) |
| 1 | 52 (26.4%) | 34 (28.6%) |
| 2 | 76 (38.6%) | 35 (29.4%) |
| 3 | 61 (31.0%) | 44 (37.0%) |
| **PCI revascularization** | 173 (87.8%) | 105 (88.2%) |
| **Stenting :** |  |  |
| LMCA | 17 (8.6%) | 14 (11.8%) |
| LAD | 93 (47.2%) | 40 (33.6%) |
| LCX | 29 (14.7%) | 26 (21.8%) |
| RCA | 43 (21.8%) | 30 (25.2%) |
| **CABG revascularization** | 11 (5.6%) | 5 (4.2%) |
| **ICU Management** ^a^ **:** |  |  |
| Dobutamine use | 181/193 (93.8%) | 110/116 (94.8%) |
| ECMO | 54/193(28.0%) | 32/116 (27.6%) |
| IABP | 37/193(19.2%) | 22/116 (19.0%) |
| Invasive ventilation | 70/193 (36.3%) | 47/116 (40.5%) |
| Lactates (mmol/l) ^b^ | 3.3 [2.2-5.0] | 3.5 [2.2-6.3] |

^a^ 7 patients deceased in catheterization laboratory before ICU admission were excluded: 4 in the pretreatment group and 3 in the no pretreatment group

^b^ Missing data: 28 for pre-treatment group and 16 no pre-treatment group.

CABG: Coronary Artery Bypass Grafting; ECMO: Extracorporeal Membrane Oxygenation; IABP: Intra-Aortic Balloon Pump; ICU: Intensive Care Unit; LAD: Left Anterior Descending Artery; LCX: Left Circumflex Artery; LMCA: Left Main Coronary Artery; PCI: Percutaneous Coronary Intervention; RCA: Right Coronary Artery

**Table S7. Antithrombotic Management according to the pre-treatment strategy – STEMI population**

|  | **Pre-treatment with P2Y12 inhibitor** | **No pre-treatment with P2Y12 inhibitor** |
| --- | --- | --- |
|  | **(N=197)** | **(N=119)** |
| **Per angiography management** |  |  |
| P2Y12 inhibitor load ^a^ | 16 (8.1%) | 71 (59.7%) |
| Ticagrelor | 10 (5.1%) | 38 (31.9%) |
| Prasugrel | 5 (2.5%) | 20 (16.8%) |
| Clopidogrel | 1 (0.5%) | 13 (10.9%) |
| Cangrelor use | 6 (3.0%) | 19 (16.0%) |
| GPI inhibitor use | 67 (34.0%) | 39 (32.8%) |
| **During hospitalization** ^b,c^ |  |  |
| Aspirin use | 186/193 (96.4%) | 113/116 (97.4%) |
| P2Y12 inhibitor use | 177/193 (91.7%) | 103/116 (88.8%) |
| Ticagrelor | 125/193 (64.8%) | 58/116 (50.0%) |
| Prasugrel | 39/193 (20.2%) | 29/116 (25.0%) |
| Clopidogrel | 55/193 (28.5%) | 41/116 (35.3%) |
| Oral anticoagulation (VKA or DOACs) | 24/193 (12.4%) | 23/116 (19.8%) |

^a^ Patients from pre-treatment group could have received another loading dose of P2Y12 inhibitor during angiography.

^b^ 7 patients deceased in catheterization laboratory before ICU admission: 4 in the pretreatment group and 3 in the no pretreatment group.

^c^ Use was defined as at least one standard daily dose administered (75mg of aspirin, 75mg of clopidogrel, 10mg of prasugrel, 90mg*2 of ticagrelor).

DOAC: direct oral anticoagulants; VKA: vitamin K antagonist.

**Table S8. Outcomes at 30 days in the STEMI population - IPW analysis**

| **Outcome** | **Pre-treatment with P2Y12 inhibitor** | **No pre-treatment with P2Y12 inhibitor** |
| --- | --- | --- |
|  | **(N=197)** | **(N=119)** |
| **MACE at 30 days^a^** | 77 | 47 |
|  | 40.0% [33.4 - 47.4] | 40.3% [32.0 - 49.8] |
| Death from any cause at 30 days^a^ | 70 | 38 |
|  | 36.3% [29.9 - 43.6] | 32.2% [24.5 - 41.6] |
| Ischemic stroke at 30 days^a^ | 10 | 10 |
|  | 6.2% [3.3 - 11.2] | 11.1% [6.2 - 19.4] |
| Peripheric ischemia at 30 days^a^ | 10 | 5 |
|  | 6.3% [3.4 - 11.3] | 5.0% [2.1 - 11.8] |
| New MI at 30 days^a^ | 7 | 2 |
|  | 4.7% [2.3 - 9.7] | 2.1% [0.5 - 8.7] |
| Stent thrombosis definite or probable at 30 days^a,b^ | 12 | 4 |
|  | 7.7% [4.4 - 13.2] | 4.0% [1.5 - 10.7] |
| **Major bleeding (BARC3/4/5) at 30 days ^a^** | 68 | 34 |
|  | 40.8% [33.6 - 48.8] | 35.3% [26.6 - 45.7] |
| Number of patients with BARC2 bleeding within 30 days | 25 | 15 |
|  |  |  |
| Number of patients with BARC3 bleeding within 30 days | 59 | 29 |
|  |  |  |
| Number of patients with BARC4 bleeding within 30 days | 3 | 1 |
|  |  |  |
| Number of patients with BARC5 bleeding within 30 days | 6 | 5 |
|  |  |  |

^a^ Number of events - Kaplan Meier estimate [95% CI].

^b^ Pre-treatment group: 3 definite ≤24h stent thrombosis, 6 definite stent thrombosis between 24hours and 30 days and 3 probable stent thrombosis. No pre-treatment group: 1 definite ≤24h stent thrombosis, 2 definite stent thrombosis between 24 hours and 30 days and 1 probable stent thrombosis

**Figure S1. Standardized mean differences for variables included in the propensity score model**


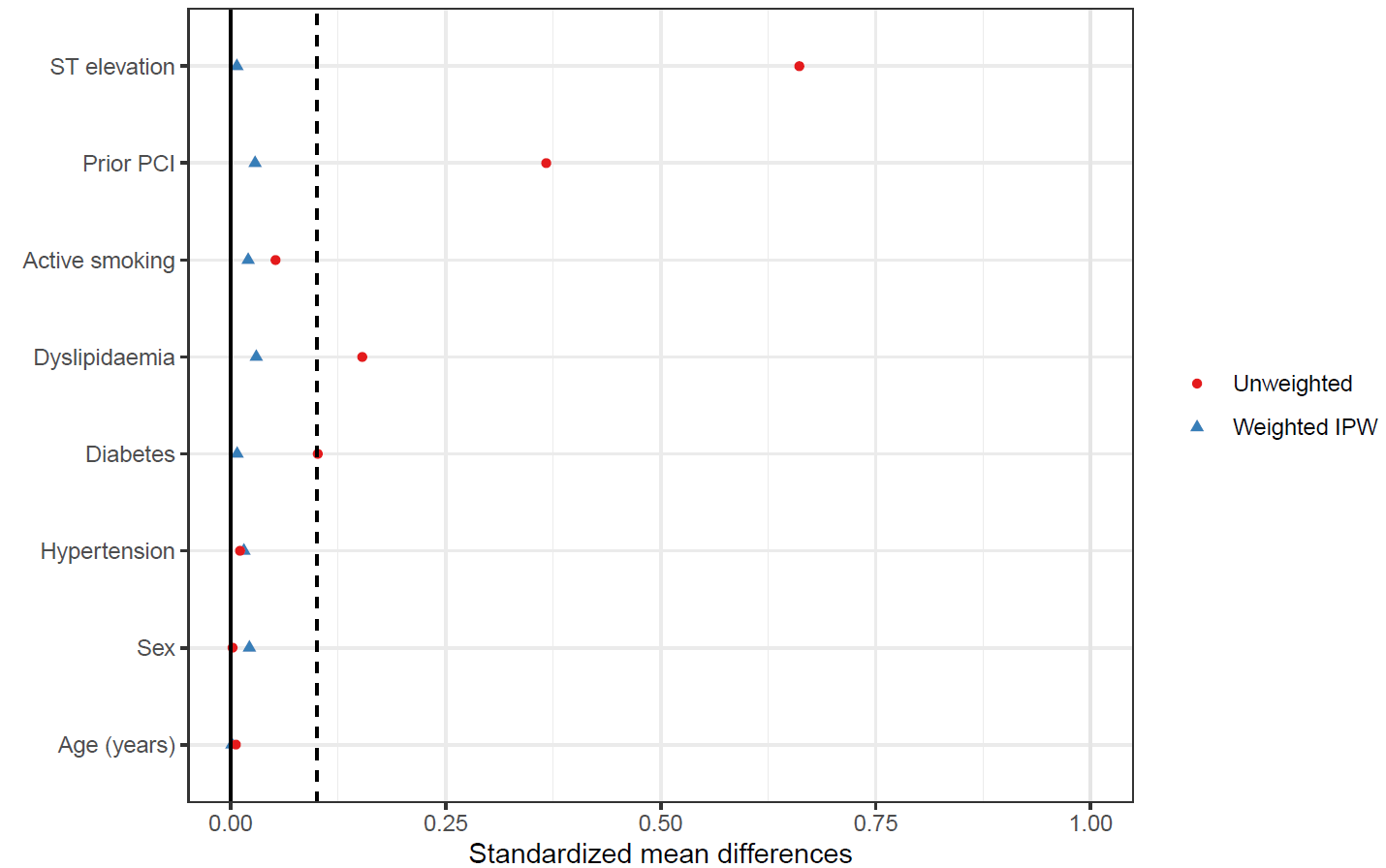


**Figure S2. Yearly Distribution of Pre-Treatment**
